# Supplementary material for: Splice-Junction-Based Mapping of Alternative Isoforms in the Human Proteome
Source: Cell Rep. Author manuscript; Available in PMC 2020 Jan 15. (PMC6961840; doi:10.1016/j.celrep.2019.11.026)
Supplement: 2 [file NIHMS1546469-supplement-2.zip › DF1/PXD009737/TestisTrypsin/TestisTrypsin_51_EIF2A_VCIFSKDGTLFAWGNGENFVRGSEGLYMVNGPPHFTESTVFPR.pdf]

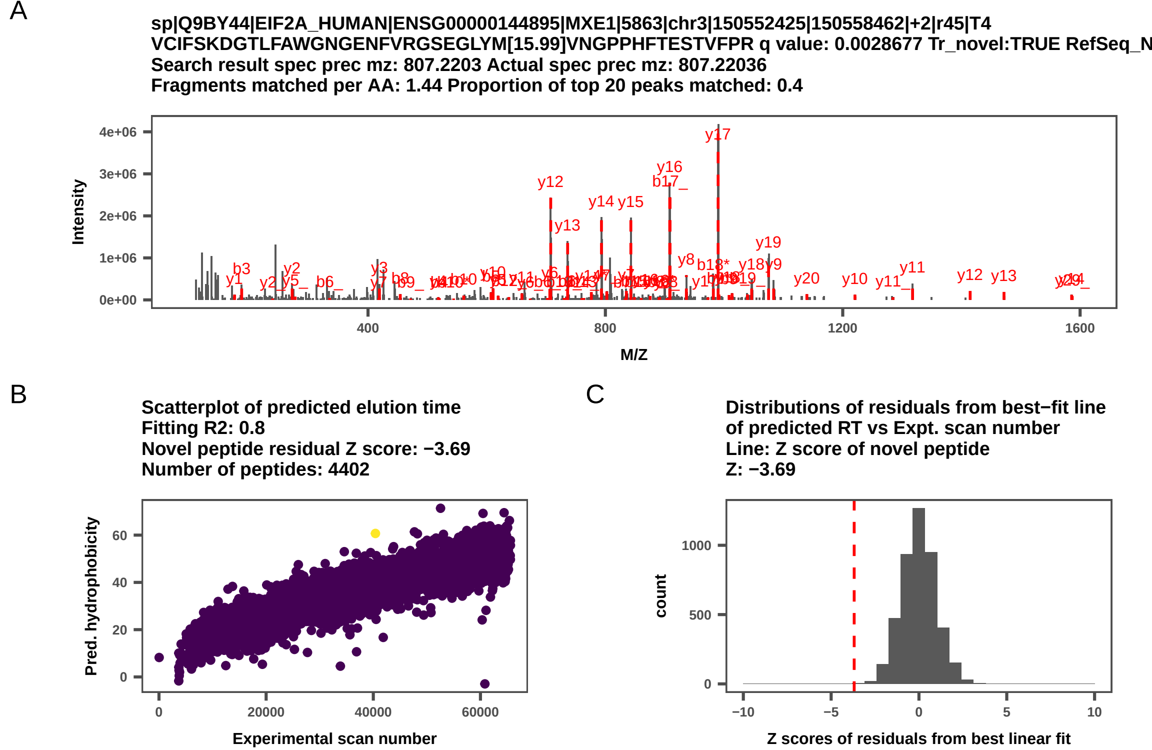
A
sp|Q9BY44|EIF2A_HUMAN|ENSG00000144895|MXE1|5863|chr3|150552425|150558462|+2|r45|T4
VCIFSKDGTLFAWGNGENFVRGSEGLYM[15.99]VNGPPHFTESTVFPR q value: 0.0028677 Tr_novel:TRUE RefSeq_N
Search result spec prec mz: 807.2203 Actual spec prec mz: 807.22036
Fragments matched per AA: 1.44 Proportion of top 20 peaks matched: 0.4
Intensity
4e+06
3e+06
2e+06
1e+06
0e+00
y17
y16
b17_
y12
y14
y15
y13
y19
y18
y9
y20
y10
y11_
y11
y12
y13
400
800
1200
1600
M/Z
B
Scatterplot of predicted elution time
Fitting R2: 0.8
Novel peptide residual Z score: −3.69
Number of peptides: 4402
Pred. hydrophobicity
60
40
20
0
0
20000
40000
60000
Experimental scan number
C
Distributions of residuals from best−fit line
of predicted RT vs Expt. scan number
Line: Z score of novel peptide
Z: −3.69
count
1000
500
0
−10
−5
0
5
10
Z scores of residuals from best linear fit
